# Effect of Intrapleural Meperidine on Post-Operative Pain after Open Cholecystectomy

**Published:** 2019-01

**Authors:** Kamran Mottaghi, Farhad Safari, Parisa Sezari, Alireza Salimi, Masoud Nashibi

**Affiliations:** Anesthesiology Research Center, Department of Anesthesiology, Shahid Beheshti University of Medical Sciences, Tehran, Iran

**Keywords:** Cholecystectomy, Pain, Intrapleural, Meperidine

## Abstract

**Background:**

Post-operative pain after open cholecystectomy can result in increased oxygen consumption, atelectasis, pneumonia, decreased vital capacity, and increased morbidity and mortality. The aim of this study was to compare the analgesic effects of intrapleural meperidine and intravenous morphine in controlling post-cholecystectomy pain.

**Materials and Methods:**

In a double-blinded randomized clinical trial, 72 patients who were candidate for elective open cholecystectomy, were divided randomly into two groups based on accidental randomized numbers. Anesthesia technique was precisely the same for all patients. At the end of surgery, 50 mg of meperidine (diluted in 20 cc normal saline) was injected intrapleurally for meperidine group patients; whereas, 0.1 mg/kg intravenous morphine was injected intravenously in control group. Onset of pain and total dose of rescue analgesic were measured.

**Results:**

In order to obtain a Numerical Rating Scale (NRS) <3, the difference in morphine consumption up to 12 hours in two groups (4.4 ±1.7 mg in meperidine group & 5±2 mg in control group) was not statistically different. However, the first request for analgesia in meperidine group was delayed significantly longer than the control group (146.6 ±6.8 minutes in meperidine group & 40 ±1.8 minutes in control group).

**Conclusion:**

A single injection of intrapleural meperidine can delay the first request for analgesia in open cholecystectomy compared to intravenous morphine.

## INTRODUCTION

Cholecystectomy is one of the most common surgeries worldwide. Early post-operative pain is the most common complaint of patients on the first day after laparoscopic or open cholecystectomy. Post-cholecystectomy pain could decrease Functional Residual Capacity (FRC), decrease vital capacity, and induce desaturation particularly in recovery time. Post-operative pain increases morbidity of patients (1).

Systemic opioids are mainstay of post-cholecystectomy analgesia in many settings, but respiratory depression, nausea, vomiting and ileus are common side effects of opioids. The high incidence of post-operative pain and risk of respiratory incompetency due to decreased FRC and vital capacity indicates the need to control this pain.

Intrapleural analgesia has been proposed as an effective method for controlling the postoperative pain, preservation of lung function after thoracotomy or cholecystectomy, and reduction of post-operative pulmonary complications (2). The mechanism of intrapleural analgesia is controversial (sensory or sympathetic block, or both). Intrapleural analgesia has been attempted in various studies; however, patients vary tremendously in their response to this block.

Meperidine is an opioid with local anesthetic effect. Intrapleural meperidine could be a safe option with higher efficacy for post-cholecystectomy pain.

Our aim was to compare the analgesic effects of intrapleural Meperidine and intravenous morphine in controlling post-cholecystectomy pain.

## MATERIALS AND METHODS

### Study design and patient selection

The study was reviewed and approved by the Shahid Beheshti University of Medical Sciences Ethics Committee and performed in accordance. Information about the study was given comprehensively both orally and in written form to all patients or their accompanying adult. They gave their informed written consents prior to their inclusion in the study.

In a randomized double blind study, 80 patients candidate for elective cholecystectomy were enrolled in this study, 8 patients were excluded due to change in the surgical technique, prolonged operation or transferring the patient to the intensive care unit (ICU) at the end of procedure. Finally, the data of 72 patients were collected. The trial was registered in the Iranian Registry of Clinical Trials under the registration number of IRCT20151012024493N3.

Inclusion criteria were patients with ASA class I and II, no addiction to drugs, no long-term analgesic use, no history of thoracotomy, age between 18 and 60 years, and Body Mass Index (BMI) between 18–30. Exclusion criteria were development of respiratory distress in recovery, duration of surgery >150 minutes, change of surgical incision other than Kocher right subcostal, and major complication of cholecystectomy procedure.

### Anesthesia

Method of anesthesia was the same in both groups. Patients were pre-oxygenated with 7 L/min 100% O_2_ for 3 to 5 min. For premedication, fentanyl 3 μg/kg and midazolam 0.03 mg/kg was administered. The anesthesia induced with propofol 2 mg/kg/IV and lidocaine 1.5 mg/kg/IV and cisatracurium 0.2 mg/kg/IV. Intubation was performed under smooth direct laryngoscopy after 5 minutes when TOF=0. Endotracheal tube size was selected after laryngoscopy under direct visualization. Anesthesia was maintained with propofol 100–200 μg/kg/min/IV, remifentanil 0.05 μg/kg/min/IV infusion and cisatracurium 0.05 mg/kg every 30 minutes and 50% Nitrous oxide/50% Oxygen to keep the depth of anesthesia between 40 and 60 on Cerebral State Monitoring (CSM).

### Groups of study:

At the end of surgery, in meperidine group, 50 mg of meperidine was diluted in 20 ml normal saline and injected into intrapleural space. In control group, intravenous morphine was injected at 0.1 mg/kg/IV. To blind the study, meperidine group received 0.1 ml/kg/IV normal saline (equal to volume of IV morphine in control group) and control group received 20 ml of intrapleural normal saline. Data collection was performed by a different physician who performed the injections.

### Intrapleural block

Intrapleural injection was performed at the end of surgery when surgical incision was closed. Patient was reversed by neostigmine (0.05 mg/kg) and atropine (0.02 mg/kg), while propofol and remifentanil infusion continued. Patient breathed spontaneously, then a Tuohy needle was applied. The technique for intrapleural block was performed with the patient in a supine position. The 5th intercostal space was identified. The needle was inserted about the midaxillary line, and an epidural needle tip was advanced until it touched the rib and then the needle walked up to rest on the cephalad edge of the rib and advanced slowly over the superior edge of the rib. When the tip of the needle entered the parietal pleura, the hanging drop was drawn into the chest cavity because of the negative intrathoracic pressure and then the prepared solution injected. Control CXR was performed to rule out pneumothorax. Then physical examination was performed to detect the subcutaneous emphysema.

**Figure 1.**
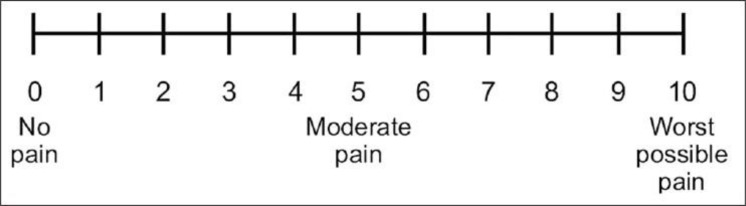
Numerical Rating Scale (NRS)

Pain score was recorded by using Numerical Rating Scale (NRS) Figure 1. Patients received intravenous morphine on their demand to keep the NRS below 3, the amount of received morphine was recorded in the first 24 hours postoperative period.

### Data Collection

The first time to request rescue morphine analgesia, total dose of administered morphine during 12 and 24 hours after surgery and NRS was recorded in prepared data sheets.

## RESULTS

A total number of 80 patients were enrolled in the study and 4 patients were excluded from each group. Age, sex, body weight and duration of surgery were not significantly different between two groups of study (P>0.05) (Table 1).

**Table 1.** Demographic variables in Meperidine and control groups

	Meperidine (n=36)	Control (n=36)	p-value
Age (years)	45.3 ± 11.7	48.1 ± 8.5	0.19^*^
Sex (Male/Female)	15/21	16/20	0.54£
Weight (kg)	76.4 ±11.6	72 ±12.8	0.17^*^
Duration of surgery (min)	89.5 ± 15	84.5 ± 12	0.21^*^

* t test; £ Chi-square

**Figure 2.**
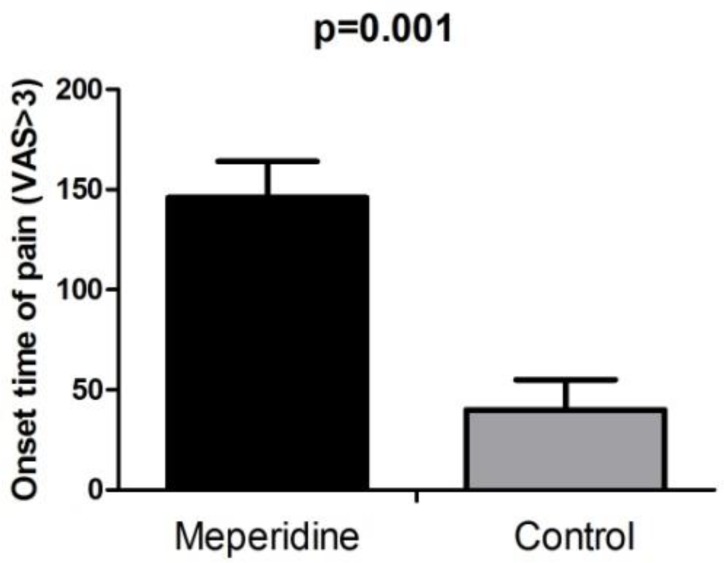
Onset time of pain (NRS >3) in Meperidine and control group.

Onset time of pain (*Visual Analogue Scale*-VAS>3) was significantly longer in meperidine group (146.6±18.5 minute) compared to control group (40.5±15.1minute) (P=0.001) (Figure 2).

Although the total dose of morphine in the next 12 and 24 hours post-operative was lower in meperidine group compared to control group (Table 2), it was not statistically meaningful.

**Table 2.** Total dose of Morphine requirement at 12 and 24 hours post-operative period in Meperidine and control groups

Post operation time	Total dose of Morphine requirement (mg)		P-value

	Meperidine (n=36)	Control (n=36)	
12 hours	4.4 ±1.79	5.4±2.32	0.8^*^
24 hours	7.5±2.52	8.9±2.61	0.14^*^

* t-test

None of patients developed pneumothorax in control CXR performed after surgery.

## DISCUSSION

In this study we assessed intrapleural meperidine versus intravenous morphine in control of the post-cholecystectomy pain. Our results showed that intrapleural meperidine is effective in deterring post-cholecystectomy pain in comparison to placebo.

Onset of pain was significantly longer in meperidine group compared to intravenous morphine. Morphine is a more potent opioid in comparison with Meperidine and has higher affinity to central μ receptors. This effect implies the fact that local anesthetic effect of meperidine in controlling post-cholecystectomy pain is superior to central effect of opioids.

Total dose of rescue morphine was not significantly different between the two groups of study. This also emphasizes that intrapleural meperidine is effective through its peripheral local anesthetic effect. Meperidine local anesthetic effect is transient and last for 3 to 4 hours, and thereafter systemic analgesic is required.

Several mechanisms have been proposed for local anesthetic-like effect of meperidine. Although less potent in comparison with lidocaine, meperidine blocks voltage-dependent Na+ channels with molecular pharmacologic features of a local anesthetic (3). Meperidine is an agonist of both μ- and κ-receptors. In addition, it was demonstrated that meperidine can block voltage-dependent Na+ channels in amphibian peripheral nerves (4). Furthermore, meperidine exerts agonist activity at the α2-adrenoreceptor, similar to clonidine and tizanidine (5). Another study showed that meperidine blocks sensory and motor nerve conduction in a dose-related manner (6).

Intrapleural analgesia has been described as a successful method for post-operative pain following open cholecystectomy and in patients who sustained several unilateral rib fractures (7). In particular it has been shown as high-quality analgesia after cholecystectomy (8) and post-thoracotomy (9). In a report by el-Naggar et al. intrapleural regional analgesia had significant prolonged pain relief requiring minimal narcotic analgesics in the first 24 hours for pain management in cholecystectomy and had significantly shortened hospital stay (10). In addition, previous clinical trial showed a superior effect of intrapleural analgesia compared to systemic analgesic on improvement of Forced Vital Capacity (FVC) on discharge (11). In a clinical trial on 34 patients, FVC and Forced Expiratory Volume in 1 second (FEV 1), which decreased markedly in the post-cholecystectomy period, improved significantly after being given intrapleural bupivacaine (12).

Intrapleural injection of local anesthetics has been debated and results from various studies that are controversial. In Strömskag et al. study, median time interval from the intrapleural injection to administration of supplementary analgesics was 260 minutes for 20 ml 0.25% bupivacaine (13).

In another study, intrapleural bupivacaine decreased post-operative pain in open cholecystectomy surgeries (14). On the contrary study, they found no subjective or objective clinical benefit of intrapleural bupivacaine for post-operative analgesia following posterolateral thoracotomy (15). Another study depicted that intrapleural administration of bupivacaine did not provide effective pain relief for ipsilateral post-thoracotomy shoulder pain (16). Besides, Strömskag et al. showed that volume of local anesthetic within the range of 20–40 mL in an adult has little influence on the extent or duration of intrapleural analgesia (13).

Although our results showed that intrapleural meperidine is an effective procedure to decrease post-cholecystectomy pain; however, further researches with higher sample size are needed to demonstrate the actual efficacy of different methods of analgesia. The main side effect of this method is minimal risk of pneumothorax (17) which we did not detect in our cases. The main side effects of opioids such a nausea, vomiting, constipation or urinary retention were not reported as well. Subcutaneous methylnaltrexone showed its efficacy in controlling some of these side effects (18).

## CONCLUSION

In conclusion, intrapleural meperidine is an effective treatment for post-cholecystectomy pain which significantly delays onset of pain in these patients. This method could be a suitable alternative in post-cholecystectomy pain control.
